# STOP— a training intervention to optimise treatment for smoking cessation in community pharmacies: cluster randomised controlled trial

**DOI:** 10.1186/s12916-022-02412-2

**Published:** 2022-06-28

**Authors:** Sandra Jumbe, Vichithranie W. Madurasinghe, Wai Yee James, Colin Houlihan, Samantha L. Jumbe, Tammy Yau, Florian Tomini, Sandra Eldridge, Borislava Mihaylova, Liz Steed, Ratna Sohanpal, Darush Attar, Stephanie J. C. Taylor, Chris Griffiths, Robert Walton

**Affiliations:** 1 Centre for Evaluation and Methods, Wolfson Institute of Population Health, Queen Mary University of London, Yvonne Carter Building, 58 Turner Street, London, E1 2AB UK; 2 Nuffield Department of Population Health, University of Oxford, Richard Doll Building, Old Road Campus, Roosevelt Drive, Oxford, OX3 7L UK; 3 Wolfson Institute of Population Health, Queen Mary University of London, Yvonne Carter Building, 58 Turner Street, London, E1 2AB UK; 4 Wessex Regional Genetics Laboratory, Salisbury NHS Foundation Trust, Odstock Rd., Salisbury, SP2 8BJ UK; 5 UC Davis Medical Center, 2315 Stockton Blvd, Sacramento, CA 95817 USA; 6 Asthma UK Centre for Applied Research, Wolfson Institute of Population Health, Barts and The London School of Medicine and Dentistry, Queen Mary University of London, 58 Turner Street, London, E1 2AB UK; 7 North Central London CCG (Barnet Borough), Treating Tobacco Dependence National Trainer, Laycock Street, London, N1 1TH UK

## Abstract

**Background:**

Community pharmacies serve people with high levels of tobacco-related illness, but throughput in NHS Stop Smoking Services in pharmacies remains relatively low. We investigated the effectiveness of a complex intervention to increase service uptake and retention.

**Methods:**

We randomised 60 pharmacies in England and Wales to the STOP intervention or usual practice in a pragmatic, parallel-group, controlled trial over 11 months. Smokers were blind to the allocation. The intervention was theory-based consultation skills training for pharmacy staff with environmental prompts (badges, calendars and behavioural cues). The primary outcome was the number of smokers attending an initial consultation and setting a quit date.

**Results:**

The intervention made no significant difference in setting a quit date, retention or quit rate. A total of 631 adult smokers (service users) enrolled and set a quit date in intervention pharmacies compared to 641 in usual practice pharmacies, a rate ratio of 0.75 (95% CI 0.46 to 1.23) adjusted for site and number of prescriptions. A total of 432 (68%) service users were retained at 4 weeks in intervention and 500 (78%) in usual practice pharmacies (odds ratio 0.80, 0.41 to 1.55). A total of 265 (42%) service users quit smoking at 4 weeks in intervention and 276 (43%) in usual practice pharmacies (0.96, 0.65 to 1.43). The pharmacy staff were positive about the intervention with 90% (56/62) stating that it had improved their skills. Sixty-eight per cent would strongly recommend the training to others although there was no difference in self-efficacy for service delivery between arms. Seventy of 131 (53%) service users did not complete the 6-month follow-up assessment. However, 55/61 (90%) service users who completed follow-up were satisfied or very satisfied with the service. All usual practice arm service users (*n* = 33) and all but one in the intervention arm (*n* = 27) would recommend the service to smokers.

**Conclusions:**

We found high levels of retention and acceptable quit rates in the NHS pharmacy stop smoking service. Despite pharmacy staff providing positive feedback on the STOP intervention, it made no difference to service throughput. Thus, other factors may currently limit service capacity to help smokers to quit.

**Trial registration:**

ISRCTN, ISRCTN16351033. Retrospectively registered.

**Supplementary Information:**

The online version contains supplementary material available at 10.1186/s12916-022-02412-2.

## Background

Tobacco is one of the leading causes of preventable death [1], causing more than seven million deaths each year worldwide [2, 3]. Around 1.2 million of these deaths are from environmental exposure to tobacco smoke [4]. In England, smoking killed 78,000 people in 2018, and 670,000 had increased social care needs from smoking-related illnesses [5] In Wales, where smoking accounts for one in six deaths of all adults aged 35 and over, approximately 5000 premature deaths in Wales are attributable to smoking-related diseases annually[6, 7]. Thirty-seven per cent of all deaths from respiratory disease and 27% of all deaths from cancer are attributable to smoking in high-income countries [8]. Smoking costs the UK economy almost £12 billion each year: employers £5.3 billion, societal costs £4.1 billion and National Health Service (NHS) £2.5 billion [5, 9]. Stopping smoking, however, is a very effective way of reducing the risk of tobacco-related illness and death [10]. Whilst sustained smoking from early adulthood may triple age-specific mortality rates, cessation at age 50 halves the risk, and those who stop aged 30 can avoid almost all the increased mortality due to smoking[11]. More recent work shows that smoking cessation is effective in reducing premature mortality at all ages, even for those in older age groups[12].

NHS Stop Smoking Services (SSS) were rolled out across England and Wales in 2000 aiming to provide free medication and behavioural support to help people to stop smoking[7, 13]. The NHS SSS are available from a number of providers such as GP surgeries, community pharmacies and specialist clinics. In 2018/2019, 236,175 people set a target date for quitting smoking in the service; of these, 123,800 (52.4%) stopped smoking at 4 weeks measured by self-report. However, quit rates in community pharmacies are 19%, lower than those in other settings [13, 14]. Previous studies suggest that pharmacies only target smokers perceived as likely to quit and that smoker retention is poor with about a third lost to follow-up [14, 15]. Nevertheless, the community pharmacy staff have contact with people less likely to attend more formal healthcare settings who may suffer a heavy burden of smoking-related disease [16]. Thus, optimising community pharmacy stop smoking services could help address health inequalities by reducing morbidity and mortality from tobacco use.

The Smoking Treatment Optimisation in Pharmacies (STOP) programme aimed to develop an intervention to increase throughput in the stop smoking services in community pharmacies and to improve quit rates by providing training including motivational interviewing and communication skills to pharmacy staff (STOP intervention) [17]. We conducted extensive qualitative research with both pharmacy staff and users of stop smoking services [14, 18], and took into account the factors associated with successful quit attempts and successful outcomes in other behavioural interventions [19, 20]. We incorporated the findings from research into communication strategies used in community pharmacies that were associated with successful quitting [20] and grounded the intervention in social cognitive theory [21] using validated behaviour change techniques [22]. We piloted the intervention [23] and refined it based on feedback from pharmacy workers and treated smokers [24]. In a further pilot study, we focused on optimising pharmacy staff attendance at the STOP training and developed ways of using simulated patients to assess intervention fidelity [25]. We drew from the diffusion of innovations theory to plan the implementation [18, 26].

Here, we report the evaluation of the STOP intervention in a cluster randomised controlled trial conducted in community pharmacies in east London, Coventry and South Wales. These areas have similar profiles with pockets of high deprivation and residents more likely to work in routine and manual jobs, together with higher smoking prevalence compared to the UK national average. Research on adult smoking habits indicates strong links between smoking and deprivation with those in more deprived areas more likely to smoke [27]. Optimising pharmacy-led stop smoking services in these areas could bring substantial health benefits from quitting on both individual and broader population levels. Findings from the process evaluation [28] and economic assessment will be published separately.

## Methods

### Study design

STOP was a pragmatic, cluster randomised controlled trial with community pharmacies as the unit of randomisation.

### Setting, participants and consenting

The STOP Trial was approved by the South Central—Hampshire A NHS Research Ethics Committee (REC) reference number 17/SC/0067 on 3 April 2017.

This study had two types of participants: staff working in community pharmacies (stop smoking advisors and support staff) and people who used the service (treated smokers). Eligible community pharmacies were those providing stop smoking services and were identified from lists provided by NHS service commissioners in each recruiting area.

Pharmacy owners were initially approached by email invitation, with an enclosed participant information sheet, then followed up with a phone call after a few days. For consenting pharmacies, a meeting was scheduled with the lead pharmacists and their staff (stop smoking advisors and support staff) to discuss the study in detail and obtain written informed consent for participation. Stop smoking advisors are the people who run the stop smoking service within the pharmacy including providing one to one consultation sessions with clients [25, 29]. The support staff, typically known as counter assistants, are the initial point of contact with pharmacy clients and are the point of first contact with smokers enquiring about stop smoking services.

**Fig. 1:**
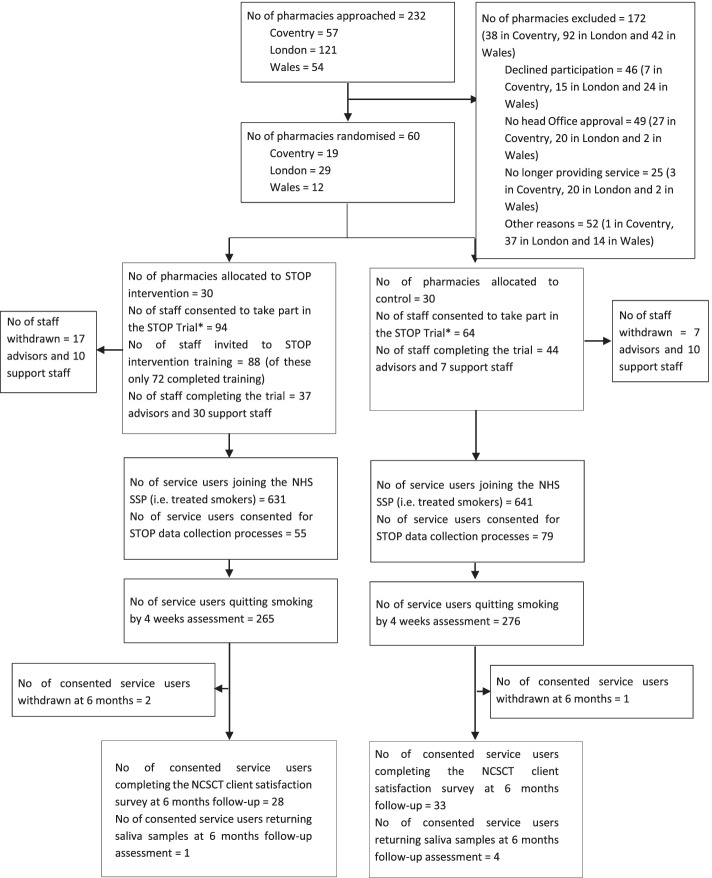
STOP Trial flowchart

Sixty community pharmacies in London (29, 24% of total eligible) and Coventry (19, 33%) in England and Cwm Taf, southeast Wales (12, 22%) agreed to participate and were randomised by cluster (see Fig. 1). Pharmacy characteristics (whether the pharmacy is a chain or independent) and pharmacy staff data (demographics, work role and experience, training needs) were collected after obtaining written consent.

All self-reported current smokers aged 18 years and older who joined the stop smoking services in participating pharmacies and attended their first stop smoking session were included in the analysis of the primary outcome. For clarity, we call this group ‘treated smokers’. Anonymised routine data were collected on treated smokers from returns to service commissioners or from manual records under the gatekeeper agreement [17]. Willing treated smokers gave individual, written, informed consent for additional data collection procedures and were contacted at 6 months to ascertain continuous abstinence status and satisfaction with NHS SSS.

### Randomisation

WYJ, SJ and TY recruited community pharmacies and emailed an independent statistician in the Pragmatic Clinical Trials Unit, who was not part of the STOP team, with relevant information. The unit of randomisation was the community pharmacy. Pharmacies were allocated to the STOP training intervention or usual practice (control group) in a 1:1 ratio using block randomisation, stratified for pharmacy commissioner and an average number of prescriptions dispensed per month per pharmacy—(1) = 0–3000 and (2) =  > 3000 based on dispensing data summaries extracted from publicly available data for England [30] and for Wales, respectively, as a proxy for pharmacy size/footfall. Three thousand was the median number of prescriptions dispensed per month per pharmacy. The independent statistician used an online randomisation system to ensure allocation concealment.

There were three types of community pharmacies: independent, small chains and large chains. To reduce contamination due to staff crossover, pharmacy chains where the owner had fewer than five pharmacies (small chains) were randomised as one unit. For large pharmacy chains, each outlet was randomised as a separate unit. Owners of large pharmacy chains or the head office confirmed to the study team at recruitment and during subsequent trial monitoring visits that there would be no staff crossover.

### Blinding

Treated smokers in both intervention and usual practice pharmacies were informed that their community pharmacy was taking part in a research study but remained unaware of the pharmacy allocation status. It was not possible to blind either participating pharmacies or pharmacy staff (i.e. advisors, support staff) to the intervention because the intervention involved staff training. The trial statistician (VM) remained blind to the allocation status until the analysis plan was signed off. Commissioners who collected and sent electronic routinely collected SSS data were also blinded to the allocation status.

### Outcome measures

#### Primary outcome

The primary outcome measure was treated smoker throughput assessed using routinely collected data supplied by service commissioners, defined as the number of smokers who joined the stop smoking service, set a firm quit date and attended at least one consultation on or before the quit date (a ‘treated smoker’ according to the Russell standard) [31].

#### Secondary outcomes

The following are the secondary outcomes:

- Four-week retention rate, defined as the proportion of treated smokers retained at 4 weeks.
- Four-week quit rate, defined as the proportion of treated smokers who quit smoking at 4 weeks from the set quit date (i.e. a ‘carbon monoxide (CO)-verified 4-week quitter’). National guidance recommends that pharmacy staff determine quit status at 4 weeks in at least 85% of cases, but there is no guidance on the number of attempts allowed to follow up treated smokers [16].
- Continuous abstinence, defined as the proportion of treated smokers who quit at 4 weeks (CO-verified) who remained abstinent at 6 months (ascertained by telephone interview and verified by salivary cotinine), assessed in a subgroup of individually consented smokers who quit at four weeks (CO-verified).
- STOP intervention acceptability (intervention arm only), assessed by a previously developed Likert scale questionnaire completed by participating staff 5 months post-consent date [23].
- Pharmacy staff self-efficacy to deliver the service.
- Treated smoker satisfaction of pharmacy-led SSS, assessed using the National Centre for Smoking Cessation and Training (NCSCT) client satisfaction survey [17]. All treated smokers providing informed consent for additional data collection procedures were contacted by a researcher at 6 months to complete this survey by telephone or by post with instructions to complete and return to the study team in freepost envelope provided. Three attempts to contact were made before a treated smoker was marked as lost to follow-up.

Methods for assessing intervention fidelity and economic assessment are published elsewhere [17, 25, 28].

### Data collection

Routine data from London and Coventry were obtained electronically from commissioners. Only pharmacy-level aggregated routine data were available from Wales; therefore, the study team manually collected and transcribed paper-based individual treated smoker data onto the electronic database at each participating pharmacy.

#### Intervention

A programme theory based on findings from our literature reviews, qualitative and pilot studies was developed to inform intervention development [14, 17–20, 23–25]. Guided by the COM-B framework for behaviour change[22], we drew upon social cognitive theory and self-determination theory to target capability and motivation among pharmacy staff [24]. The assumption was that addressing pharmacy workers’ skills, attitudes and motivation towards smoking cessation through practice-based training sessions would lead to more effective engagement of smokers and better quit rates in the stop smoking service [24, 25].

The original design was for training to be delivered over two sessions with opportunities to practice skills learnt during training in between. However, based on feedback from pharmacy workers who took part in pilot studies, the STOP training intervention was offered as a face-to-face, single, off-site, half-day session on a pharmacy closure day (Saturday or Sunday), lasting approximately 3 h [23–25]. Pharmacy stop smoking advisors and support staff (e.g. counter assistants) were trained together to encourage team working and to facilitate role modelling by more experienced staff. The STOP training involved role-plays and videos targeting the engagement of smokers and optimising the delivery of smoking cessation counselling. The training also focused on communication skills based on motivational interviewing and practising key phrases such as ‘all quit attempts are a success’, ‘our service is free, delivered with an expert’ and ‘you can come back anytime for support’, to facilitate better engagement with potential SSS clients [24, 25]. Training sessions were facilitated by a health psychologist experienced in behaviour change (SJ), an experienced respiratory nurse (WYJ) and a community pharmacy stop smoking adviser (DA). We chose venues commonly used for other pharmacy staff training courses (i.e. existing pharmacy training rooms, university premises, local community health centres or local council premises). We established a WhatsApp group to facilitate contact between the staff after the training sessions. Training attendance was incentivised by providing £40 to counter assistants and £80 to pharmacists/stop smoking advisors to cover time spent and travel expenses (Additional file 1: Fig. S1).

Attendees were given a certificate of completion, alongside training handouts, badges, a STOP poster and a specially designed desk calendar to act as prompts and reinforcers of the training within the pharmacy environment[25]. Badges were intended to be worn by the staff during the working day and the desk calendars to be kept in the pharmacy consulting room. WhatsApp groups were set up for communication, signposting training materials and other smoking cessation resources to participants. The control arm also had a WhatsApp group solely for communication purposes.

STOP training is complementary to the National Centre for Smoking Cessation and Training (NCSCT) [29], mandatory training for stop smoking advisers. NCSCT training is a competence-based programme aiming to impart knowledge and skills in smoking cessation. The support staff had no previous smoking cessation training since only stop smoking advisors are eligible for the NCSCT programme.

#### Control group

Control pharmacies did not receive STOP intervention training and continued to deliver the NHS SSS as usual. In theory, stop smoking advisers from pharmacies commissioned to deliver NHS SSS would have had NCSCT equivalent skills-based training (possibly over 2 days) that would cover a large element of behavioural support and recruitment in addition to the online NCSCT core knowledge and skills online assessment. The STOP training aimed to bolster this among NCSCT-trained stop smoking advisers and introduce cessation concepts to untrained staff within intervention pharmacies.

Support typically provided by pharmacy-led NHS SSS involved:

- Free behavioural support and advice with an expert stop smoking advisor for 12 weeks. The first 4 weeks include weekly one-to-one in-person appointments with the advisor, followed by less frequent check-ins by phone text or email [32].
- Stop smoking medication, i.e. nicotine replacement therapy (free to those eligible for free prescriptions).
- Carbon monoxide checks at each appointment to monitor progress.

### Sample size

Because the primary outcome was at the level of the pharmacy, we conducted the sample size calculation at the pharmacy level. On average, 165 smokers were expected to join stop smoking services in a control cluster pharmacy over 11 months (based on pilot data) [23]. We considered that an increase of 33% in the intervention clusters to 220 smokers per pharmacy would be sufficient to change commissioning policy. Based on historical service commissioner data from the pilot study [23] and NHS dispensing data for pharmacies [30], the standard deviation of throughput was expected to be approximately 63 in both groups. To detect this difference at the 5% significance level and 90% power, we needed 56 clusters. We added two clusters to each arm to allow for dropout, which increased the number of community pharmacies required to 60.

### Statistical analysis

Analysis was performed on intention-to-treat (ITT) principles, where all pharmacies were included for analysis in the trial arm to which they were randomised. Data from pharmacies and treated smokers who withdrew were included in the analysis up to the point of withdrawal. By definition, there was no withdrawal for the primary outcome.

An estimate of treatment effect on the primary outcome (i.e. the numbers of smokers enrolled in the stop smoking services) was obtained by fitting a Poisson regression model with numbers of ‘treated smokers’ per pharmacy as the dependent variable. The two stratification factors, pharmacy commissioner and average number of prescriptions (i.e. forms) per month (3000 or less, more than 3000) which is a proxy for a number of smokers using stop smoking services, were included as categorical variables. Pharmacy was included as a random effect to allow for clustering.

Four-week retention and quit rates (CO-verified) for treated smokers were assessed using generalised estimating equations which included these as the dependent variable in the xtgee command in Stata with a logit link. Again, pharmacy was included as the random effects variable to account for clustering. Other covariates included in the models were two stratification factors—pharmacy commissioner and the average number of prescriptions/forms per month (3000 or less, more than 3000)—gender and age. Exchangeable correlation matrix and robust standard errors were used.

The number and percentage of treated smokers who quit (CO-verified) smoking by their 4 weeks quit status assessment and remained so at 6 months (continuous abstinence at 6 months) from the date of consent were summarised descriptively (due to the small number treated smokers (*n* = 5) providing data, no statistical modelling was performed) by intervention arm.

Stop smoking advisor and pharmacy support staff self-efficacy in delivering stop smoking services, acceptability of the STOP training intervention (applicable to intervention arm only) and treated smoker satisfaction with stop smoking services (collected from consented treated smokers at 6 months follow-up) were summarised descriptively by intervention arm. Changes in stop smoking advisor overall self-efficacy and self-efficacy in delivering core competency skills were assessed using longitudinal linear mixed effects models. Pharmacy and advisor were included as random effects to allow for clustering. Other covariates included in the models were two stratification factors—pharmacy commissioner and number of prescriptions/forms per month (3000 or less, more than 3000).

### Patient and public involvement

Public representatives, commissioners, pharmacy owners and pharmacy workers were involved in shaping the research question and designing the STOP programme from inception. Participant information sheets and consent forms were reviewed by public representatives and modified in response to their comments. We kept the public, public health commissioners and local pharmacy community informed about publications and events using the STOP Trial Twitter feed.

## Results

### Baseline characteristics

Pharmacy and smoker recruitment and follow-up are summarised in Fig. 1. We invited 232 community pharmacies, and 109 expressed an interest to be involved. We recruited 60 pharmacies from that pool over 10 months from April 2017 to February 2018, and half were randomised to the STOP intervention arm. The intervention arm had more large chain pharmacies than the control arm (Table 1). Nearly half of the recruited pharmacies had only one trained stop smoking advisor. Two-thirds of consenting pharmacy staff were stop smoking advisers; the remainder were support staff. Fewer support staff consented to participating in the trial in the control arm compared to the intervention arm (20% vs 43%). In 19 control and 11 intervention pharmacies, no support staff were recruited for the trial (Table 1).

**Table 1:** Baseline characteristics of participating pharmacies

Characteristics	Allocation				Total	
	**STOP training intervention**		**Control**			
	**No**	**%**	**No**	**%**	**No**	**%**
**Total**	**30**	**100.00**	**30**	**100.00**	**60**	**100.00**
**Area**						
NHS Barking and Dagenham CCG	3	10.00	3	10.00	6	10.00
NHS City and Hackney CCG	2	6.67	1	3.33	3	5.00
NHS Islington CCG	2	6.67	3	10.00	5	8.33
NHS Newham CCG	4	13.33	4	13.33	8	13.33
NHS Tower Hamlets CCG	3	10.00	4	13.33	7	11.67
Cwm Taf University Health Board	6	20.00	6	20.00	12	20.00
Coventry City Council	10	33.33	9	30.00	19	31.67
**Average no. of prescriptions per month**						
3000 or less	14	46.67	13	43.33	27	45.00
More than 3000	16	53.33	17	56.67	33	55.00
**Type of pharmacy**						
Independent (one pharmacy only)	9	30.00	12	40.00	21	35.00
Small chain (two to five pharmacies)	2	6.67	4	13.33	6	10.00
Multiple contractor (six or more pharmacies)	19	63.33	14	46.67	33	55.00
**Number of smoking advisors consented per pharmacy**						
1	14	46.67	15	50.00	29	48.33
2	10	33.33	11	36.67	21	35.00
More than 2	6	20.00	4	13.33	10	16.67
Mean (sd)	54^a^	1.8 (0.93)	51^a^	1.7 (0.88)	105^a^	1.8 (0.90)
Median (iqr)	54^a^	2 (1 to 2)	51^a^	1.5 (1 to 2)	105^a^	2 (1 to 2)
**Number of support staff consented per pharmacy**						
0	11^b^	36.67	19^c^	63.33	30	50.00
1	6	20.00	10	33.33	16	26.67
2	8	26.67	0	0.00	8	13.33
More than 2	5	16.67	1	3.33	6	10.00
Mean (sd)	40^a^	1.33 (1.35)	13^a^	0.43 (0.68)	53^a^	0.88 (1.15)
Median (iqr)	40^a^	1 (0 to 2)	13^a^	0 (0 to 1)	53^a^	0.5 (0 to 1)
**Number of pharmacy staff consented per pharmacy**						
1	5	16.67	7	23.33	12	20.00
2	9	30.00	17	56.67	26	43.33
3	4	13.33	3	10.00	7	11.67
4	7	23.33	1	3.33	8	13.33
5	2	6.67	2	6.67	4	6.67
More than 5	3	10.00	0	0	3	5.00
Mean (sd)	94^a^	3.13 (1.83)	64^a^	2.13 (1.04)	158^a^	2.63 (1.56)
Median (iqr)	94^a^	3 (2 to 4)	64^a^	2 (2 to 2)	158^a^	2 (2 to 3.5)

^a^Number of staff (i.e. stop smoking advisors/support staff)

^b^Four pharmacies from Wales, 5 pharmacies from Coventry and 2 pharmacies from London did not consent any support staff

^c^Five pharmacies from Wales, 6 pharmacies from Coventry and 8 pharmacies from London did not consent any support staff

Treated smoker recruitment began in July 2017 after the first pharmacy was randomised and was closed in March 2019. Control arm pharmacies had 11 months to recruit smokers from the date of randomisation. Intervention arm pharmacies had 11 months from the date of STOP Training completion.

A total of 1272 treated smokers enrolled and set a quit date (throughput) with 631 in intervention and 641 in the control arms (Table 2). Just 11% of smokers consented to provide additional data on quit outcomes at 6 months together with saliva samples for DNA extraction and nicotine metabolic rate calculation (Fig. 1). Gender and mean age of treated smokers were evenly balanced between the two arms (Table 2). However, a greater proportion of treated smokers in the intervention arm were not entitled to free prescriptions (73% vs 59% in the usual practice arm).

**Table 2:** Characteristics of treated smokers in STOP intervention and control pharmacies

Treated smoker characteristics	Allocation				Total	
	**STOP training intervention**^a^		**Control**^b^			
	***N***	**%**	***N***	**%**		
**Total**	**631**	**100.00**	**641**	**100.00**	**1**	**100.00**
**Area**						
NHS Barking and Dagenham CCG	25	3.96	43	6.71	68	5.35
NHS City and Hackney CCG	53	8.34	21	3.28	74	5.82
NHS Islington CCG	25	3.96	67	10.45	92	7.23
NHS Newham CCG	207	32.81	90	14.04	297	23.35
NHS Tower Hamlets CCG	115	18.23	68	10.61	183	14.39
Coventry	101	16.01	182	28.39	283	22.25
Wales	105	16.64	170	26.52	275	21.62
**Gender**						
Female	320	50.71	314	48.99	634	49.84
Male	311	49.29	327	51.01	638	50.16
**Age (in years)**						
Age, mean (sd)	628	46.10 (14.07)	641	45.56 (14.56)	1269	45.83 (14.31)
Age, median (iqr)	628	46 (35 to 57)	641	45 (34 to 56)	1269	46 (35 to 57)
Missing	3	–	–	–	–	–
**Entitled to free prescriptions**						
Yes: exempt from charges	171	27.10	258	40.25	429	33.73
No: pay for prescriptions	459	72.74	381	59.44	840	66.04
Missing	1	0.16	2	0.31	3	0.24
**Self-reported number of cigarettes smoked per day *(collected when smokers enrol on NHS SSS)***						
10 or less	137	21.73	130	20.28	267	20.99
11–20	313	49.60	215	33.54	528	41.51
21 or more	119	18.86	154	24.03	273	21.46
Missing	62	9.83	142	22.15	204	16.04
Mean (sd)	569^c^	17.20 (8.56)	499^c^	18.19 (9.88)	1068^c^	17.66 (9.21)
**Self-reported numbers of years smoked *(collected when smokers enrol the NHS SSP)***						
< 1 year	61	9.67	6	0.94	67	5.27
1–2 years	7	1.11	12	1.87	19	1.49
3–4 years	13	2.06	19	2.96	32	2.52
5–6 years	16	2.54	13	2.03	29	2.28
7–8 years	26	4.12	17	2.65	43	3.38
9–10 years	46	7.29	42	6.55	88	6.92
11–15 years	55	8.72	55	8.58	110	8.65
16–20 years	103	16.32	60	9.36	163	12.81
21–30 years	106	16.80	84	13.11	190	14.94
31–40 years	50	7.92	82	12.79	132	10.38
41–50 years	59	9.35	39	6.08	98	7.70
> 50 years	19	3.01	33	5.15	52	4.09
Missing	70	11.09	179	27.93	249	19.58

^a^Total number of prescriptions over the trial period in the STOP training intervention arm is 1,367,397

^b^Total number of prescriptions over the trial period in the control arm is 1,250,774

^c^Number of treated smokers

### Primary outcome: treated smoker throughput

Throughput was less than expected with on average only 21 treated smokers per pharmacy setting a quit date over the period. Our expected average was 165 smokers per control cluster pharmacy over an 11-month period. There was no difference in the number of smokers joining and setting a quit date (i.e. throughput) in the intervention arm (*n* = 631) compared to the control arm (*n* = 641); the incidence rate ratio was 0.75 (95% CI, 0.46 to 1.23) when adjusted for the site and number of prescriptions at baseline (Table 3). Of the 60 pharmacies randomised, 6 intervention and 1 control pharmacies did not enrol any smokers during the 11-month trial period. Disruptions to service delivery due to contract changes affected treated smoker recruitment in Coventry interrupting services in 3 intervention and 1 control pharmacies.

**Table 3:** Analysis of primary outcome: treated smoker throughput in the STOP intervention and control pharmacies

**Trial arm [number of pharmacies]**	**Total throughput**	**Throughput per pharmacy [mean and (sd)]**	**Incidence rate ratio**^c^** [95% confidence interval]**	***p*-value**
Control (30)^a^	641	21.37 (20.04)	Ref	
Intervention (30)^b^	631	21.03 (23.30)	0.75 (0.46 to 1.23)	0.259

Intra-cluster correlation coefficient = 0.026 (95% CI 0.001 to 0.052)

^a^One (from Coventry) of 30 pharmacies did not enrol any smokers to the NHS SSP

^b^Six (3 from Coventry, 1 from London and 3 from Wales) of 30 pharmacies did not enrol any smokers to the NHS SSP. Of these 6, 1 had withdrawn from the STOP Trial

^c^Adjusted for site and number of prescriptions/forms per month category. Number of prescriptions/forms per pharmacy over the 11 months of the trial (identified by the trial start and end dates for each pharmacy) was included as the offset

### Secondary outcomes

#### Retention rate and quit rate at four weeks

There was no significant difference in the retention rates or quit rates at 4 weeks between the STOP intervention and control pharmacies (Table 4). A total of 432 (68.5%) treated smokers were retained at 4 weeks in intervention pharmacies and 500 (78.0%) in control pharmacies (odds ratio 0.80, 0.41 to 1.55). A total of 265 (42.0%) treated smokers quit at 4 weeks in the intervention arm and 276 (43.1%) in control pharmacies (0.96, 0.65 to 1.43) which is comparable to the average CO-validated NHS SSS quit rate in England of 36%, and considered acceptable [13].

**Table 4:** Analysis of secondary outcomes: retention rate and quit rate at 4 weeks

**Outcome**	**STOP training intervention**		**Control**		**Odds ratio (95% confidence interval)**	***p*-value**	**Intra-cluster correlation coefficient**
	**Number of pharmacies**	**Number of quit attempts (%)**	**Number of pharmacies**	**Number of quit attempts (%)**			
Treated smoker retention rate in 4 weeks assessment	24	432/631 (68.46%)	29	500/641 (78.00%)	0.80 (0.41 to 1.55)	0.509	0.179
Quit rate at 4 weeks assessment	24	265/631 (41.99%)	29	276/641 (43.06%)	0.96 (0.65 to 1.43)	0.856	0.059

#### Continuous abstinence at 6 months

Only 55 and 79 treated smokers from the intervention and control arms, respectively, consented to provide additional quit outcome data and saliva samples at 6 months (Fig. 1). Of these 134 consenting treated smokers, 61 (28 from the intervention arm) were contactable for their 6 months follow-up data collection. Three treated smokers withdrew their consent when contacted at 6 months, and only 5 (1 from the intervention arm) provided saliva samples.

#### Treated smoker satisfaction

Treated smoker satisfaction was high in both arms. Thirty-two of 33 from the control arm and 23/28 from the intervention arm who completed their 6 months follow-up were either satisfied or very satisfied with the NHS SSS they received (Additional file 2: Table S1). Moreover, apart from one person from the intervention arm, all treated smokers from both arms would recommend the service to other smokers (Additional file 2: Table S2). However, it is worth noting that 70/131 (53%) treated smokers were lost to follow-up, i.e. did not complete their 6-month follow-up assessment.

#### Acceptability of the intervention to pharmacy staff

Of the 94 intervention trained staff, 30/54 advisers (56%) and 32/40 support staff (80%) provided feedback on intervention acceptability (Additional file 2: Tables S3 and S4). These 62 staff found the intervention highly acceptable. Most advisers and support staff found the training useful (31%) or very useful (61%), enjoyable (65%) and felt that it had improved their skills (90%). Only one adviser out of 30 who responded felt the training was not useful, and one other felt indifferent. Sixty-eight per cent (23 support staff and 19 advisers) would strongly recommend the training to others, but only 50% strongly felt that they were able to implement skills learnt in practice. Two support staff felt the support materials (badges, desk calendar, etc.) were not useful, and three additional staff were indifferent. The rest (92%) felt the materials were useful (*n* = 24) or very useful (*n* = 33). Seventy-three per cent (26 advisers; 19 support staff) felt able to use WhatsApp for communication easily as part of the intervention.

#### Self-efficacy of pharmacy workers in service delivery

Perceived self-efficacy to deliver SSS at baseline and 5 months post-trial consent is presented in Additional file 2: Tables S5 and S6. All staff (105 advisers and 53 support staff) consented to the STOP Trial and completed self-efficacy questionnaires at baseline. There were no significant differences in advisor self-efficacy scores between the two arms (Additional file 2: Table S5). Due to high staff withdrawal rates (30.1%), self-efficacy questionnaire completion rates at 5 months post-trial were low for support staff; no further analysis was performed (Additional file 2: Table S6).

#### Pharmacy staff and treated smoker withdrawals

Twenty-seven out of the 94 (29%) consented pharmacy staff (17 advisors and 10 support staff) from the intervention arm withdrew over the trial duration compared to 17 (7 advisors) of the 64 (26.5%) consented staff from the control arm. Of the 134 treated smokers consented in the STOP Trial, only 3 actively withdrew from the trial at their 6-month follow-up. However, over half of the 134 treated smokers were not contactable at 6 months (Table 1).

No adverse events, e.g. death, reaction to nicotine replacement therapy (NRT), were reported.

## Discussion

### Primary outcome

The STOP Trial aimed to find out whether a behaviour change and communication skills intervention to complement NCSCT training could improve throughput in community pharmacy NHS Stop Smoking Services. Although the intervention was comprehensive, theoretically grounded [24, 33] and well-received, we found no evidence that the STOP intervention increased throughput.

We consider several reasons why there was no difference in throughput between the two arms, despite pharmacy staff finding the STOP training acceptable and useful. Firstly, from 2017, budgets for services were cut by 50%[34]. These funding cuts have forced local authorities to decommission many SSS in community pharmacy settings [35, 36]. During our study period, we observed several changes in contracting processes and significant staff turnover which interrupted service activity. These changes and uncertainties in staffing and regarding service commissioning may have affected the capability and motivation of pharmacy staff to offer stop smoking services and implement the intervention. More details will be available in our forthcoming process evaluation [28]. Secondly, fewer smokers joined the service over the course of the trial recruitment period than we expected based on our pilot trial and historical commissioner data [23]. For example, from 2016/2017 to 2018/2019, the number of people approaching pharmacies decreased from 19.1 to 18.5%. In the context of the programme theory for the STOP intervention [15, 24], reduced smokers visiting pharmacies and signing up to the SSS gave pharmacy staff less opportunities to practice knowledge and skills learnt during training [14]. A recent review found that new skills acquired through training, require considerable practice time to embed those skills in day-to-day clinical practice [37]. The study also found pharmacies to be particularly challenging settings for behaviour change [37]. The process evaluation [28] will provide a more in-depth understanding of the challenges in the pharmacy context.

Another reason for the general low throughput in pharmacies during the trial may be the increasing use of electronic cigarettes (e-cigarettes) in the UK [27, 38]. E-cigarette use among adults in Great Britain between 2017 and 2019 (study period) increased from 2.9 million to 3.6 million users [39]. E-cigarettes entered the market in the UK in 2007 [40], and these devices may help some smokers quit smoking [41, 42]. During our study period, smokers may have independently tried e-cigarettes rather than accessing support from the NHS SSS. E-cigarettes have been cited as a reason for the decline in services but to a lesser extent than budget cuts [43]. Up to 2015, however, there was no clear association found between e-cigarette use and change in behavioural support to quit, but smokers trying to quit with e-cigarettes were less likely to obtain NRT on prescription [44].

### Secondary outcomes

The STOP intervention was highly acceptable to pharmacy staff, who found the sessions useful and enjoyable and felt that it improved their skills in helping people to stop smoking. This suggests that the STOP intervention model may be effective in facilitating the development of behaviour change skills in pharmacy staff in the context of smoking cessation and could therefore be useful for other health promotion initiatives. Unfortunately, we were unable to assess stop smoking adviser behaviour post-training because they were unwilling to record their smoker consultations. Moreover, 50% of staff felt that it was hard to implement skills learnt in practice. So, there are questions regarding the fidelity of intervention delivery that will be addressed in the process evaluation paper. Levels of self-efficacy for smoker recruitment, engagement and retention were high among staff in both arms of the trial suggesting that the workforce has a high level of belief in their ability to perform these tasks which may increase their motivation to engage with smokers. Recruitment and retention of smokers in the service were high at 4 weeks, and quit rates were acceptable in both study arms. Taken as a whole, we do not know whether the STOP intervention improved the behaviour change skills of pharmacy staff enough to increase throughput in the SSS. The process evaluation [28] will provide a more in-depth understanding of the challenges to the fidelity of intervention delivery.

### Comparison with other studies

To our knowledge, this is the first trial focusing on improving smoker uptake and retention in NHS community pharmacy stop smoking services and one of the few randomised controlled trials conducted in community pharmacies in the UK.

Another study that developed a behaviour change app (StopApp) to optimise uptake and attendance of NHS Stop Smoking Services more generally, also reported declining service uptake in recent years [45]. StopApp used the Capability Opportunity Motivation Behaviour (COM-B) behaviour change framework which also informed the STOP intervention. We do not know if the StopApp was successful because the trial results are not yet published. However, the StopApp feasibility trial identified several barriers to attending SSS, including lack of knowledge about what happens in the SSS (capability), the belief that the SSS is not easy to access (opportunity), that there would be ‘scare tactics’ or ‘nagging’, and no knowledge of success quit stories (motivation) [45]. These barriers are similar to those we found when developing the STOP intervention and which our intervention was designed to circumvent. We trained the pharmacy staff to proactively promote their service structure to smokers from the counter to increase knowledge (capability), encourage an ‘open door’ policy (opportunity) and approach each smoker and peers in a non-judgmental positive mindset to facilitate change (motivation) [24, 25].

### Strengths of this study

The STOP intervention was theory-based and piloted and repiloted during the course of development. We involved smokers, pharmacies and service commissioners from an early stage as part of patient and public involvement, using their feedback to adapt the content and mode of delivery to suit the community pharmacy environment. The STOP intervention was well received, and the pharmacy staff found it useful in improving their behaviour change skills. Thus, the methods and programme theory underlying the intervention may be useful in the future as more clinical tasks are taken up by pharmacists and new services are developed. Similarly, the methods developed for data analysis and evaluation of outcomes may be helpful in the design and evaluation of new programmes for health promotion in community pharmacies.

Using routinely collected data directly from service commissioners to assess smoker throughput and quit rates in the trial was a novel approach, done once before in the ‘Evaluating longer-term outcomes of the National Health Service (NHS) Stop Smoking Services’ (ELONS) study [46]. This approach reduced the additional burden of collecting data on stop smoking advisors in busy community pharmacies [23, 47]. Routinely collected data has substantial potential to improve conduct and reduce costs of RCTs although it is important to address potential practical barriers such as location and access to routine data, availability of IT infrastructure, and data quality assessment. Routine data trials also present different issues regarding informed consent, confidentiality risk management in the regulatory and ethical approval process [47].

We achieved our recruitment targets for community pharmacies and pharmacy staff using a hierarchical or ‘Russian doll’ approach [48], where the study team sought approval from high-level decision-makers (SSS commissioners, pharmacy owners) first before approaching the individual pharmacies and managers about trial participation.

### Limitations

Fewer people were enrolled in the pharmacy-led SSS than anticipated over the trial duration which limited the participant recruitment pool. Midway through the STOP Trial, pharmacy-led SSS had reduced to such an extent that we had approximately the same number of smokers using the services from 60 pharmacies over 11 months as we had from 12 pharmacies over 5 months in the pilot trial [23]. On average, only 21 smokers joined the stop smoking services per pharmacy instead of the expected 165. This decline occurred within a very short period, limiting our ability to make contingency plans. The reasons for this rapid decline are uncertain. However, cuts to the public health funding and to wider local authority spending in recent years have had a major impact on local authority budgets for stop smoking services and wider tobacco control. In 2018, 38% of local authorities in England that still had a budget for stop smoking services cut this budget, following similar cuts in 50% of local authorities in 2017 and in 59% of local authorities in 2016 [34].

The power calculation for our study was made at a cluster level based on average recruitment rates from historical data and from our pilot trial. Whilst smaller numbers would not affect the power of our study directly, this decrease in recruitment was not uniform across pharmacies, thus greater variability gave less power to detect the 33% increase in throughput.

Pharmacy withdrawals and staff turnover were high which limited data collection on self-efficacy at follow up. Participating pharmacy staff lacked experience in conducting clinical trials and needed additional training in Good Clinical Practice (GCP), informed consent and sample collection. This affected number of smokers consenting for additional data collection at 6 months and resulted in low numbers of baseline saliva samples. Pharmacy staff were resistant to the saliva collection process as they felt it would deter clients from joining their SSS. These factors limited our ability to validate self-reported quit rates in a subgroup of participants as we had intended. The lack of research readiness in community pharmacies will need to be addressed in future clinical trials in this setting.

Treated smokers provided contact details on their consent forms but very few responded to emails from STOP researchers. Those who did respond and completed a 6-month assessment were not willing to provide saliva sample. Very few were also willing to participate in an interview regarding their experience of using the service. Low response rates limited evaluation of long-term outcomes/abstinence. An explanation might be that researchers were too detached or distanced from the treated smoker group from the onset as they were recruited to the trial by adviser. More proactive communication by the study team after consent might have improved follow up rates.

## Conclusions

Within the STOP Trial, we found high levels of retention and acceptable quit rates in the NHS pharmacy stop smoking service. Pharmacy staff found the STOP training helpful in improving behaviour change skills, and service users were highly satisfied despite no increase in service throughput being detected among participating community pharmacies in the trial’s intervention arm. If health policy is to continue expanding the role of pharmacy in primary healthcare delivery, then pharmacy staff need increased levels of funding and training. UK qualitative research studies indicate enthusiasm among pharmacists for new extended primary care roles but highlight a need for clear definitions of the knowledge, skills and attributes required and provision of training that addresses these needs [14, 49].

The lack of consistent long term funding may be a potential barrier to the wider implementation of this extended role [35]. Given that participants in both trial arms had acceptable quit rates we call for commissioners to reprioritise funding into pharmacy-led SSS. Commissioners may need to consider uniformity of systems used for smoking cessation data capture to facilitate prompt remuneration to community pharmacies, and easier evaluation of outcomes on micro and macro levels across the service. Currently, the processes may be more complicated than necessary which can deter pharmacy staff from delivering the service, affecting smoker throughput. The NHS Stop Smoking Service remains an effective route for facilitating cessation if smokers can be engaged effectively.

## Supplementary Information

**Additional file 1:** **Figure S1.** TIDieR Checklist for the STOP Trial Intervention.

**Additional file 2:** **Table S1.** Treated smoker satisfaction with the NHS SSS. **Table S2.** Would you recommend this service to other smokers who want to stop smoking? **Table S3.** Acceptability of the intervention to pharmacy support staff. **Table S4.** Acceptability of the intervention to pharmacy stop smoking advisors. **Table S5.** Stop smoking advisors’ self-efficacy over time. **Table S6.** Pharmacy support staff self-efficacy over time.

## Data Availability

The lead author (SJ) affirms that the manuscript is an honest, accurate and transparent account of the study being reported. The dataset supporting the conclusions of this article is included within the article and its additional files. Anonymised trial data can be made available following a reasonable written request from the study guarantors, Professor Robert Walton (r.walton@qmul.ac.uk) and Professor Stephanie Taylor (s.j.c.taylor@qmul.ac.uk).

## Abbreviations

CO: Carbon monoxide
NCSCT: National Centre for Smoking Cessation and Training
NHS: National Health Service
NRT: Nicotine replacement therapy
REC: Research Ethics Committee
SSS: Stop smoking services
