# Supplementary material for: STOP— a training intervention to optimise treatment for smoking cessation in community pharmacies: cluster randomised controlled trial
Source: BMC Med. 2022 Jun 28;20:212. doi: 10.1186/s12916-022-02412-2 (PMC9238035; doi:10.1186/s12916-022-02412-2)
Supplement: Supplementary file 2 — Additional file 2: Table S1. Treated smoker satisfaction with the NHS SSS. Table S2. Would you recommend this service to other smokers who want to stop smoking? Table S3. Acceptability of the intervention to pharmacy support staff. Table S4. Acceptability of the intervention to pharmacy stop smoking advisors. Table S5. Stop smoking advisors’ self-efficacy over time. Table S6. Pharmacy support staff self-efficacy over time. [file 12916_2022_2412_MOESM2_ESM.docx]

**Supplementary tables**

**Table S1: Treated smoker satisfaction with the NHS SSS**

Q. Overall, how satisfied were you with the support you received to stop smoking?

response options are – Very Unsatisfied, Unsatisfied, Unsure, Satisfied, Very Satisfied

| **Allocation** | ***Number of treated smokers** | **Very unsatisfied (%)** | **Unsatisfied (%)** | **Unsure (%)** | **Satisfied (%)** | **Very satisfied (%)** | **Follow-up incomplete (%)** |
| --- | --- | --- | --- | --- | --- | --- | --- |
| Control | 78 | 0 (0.00) | 0 (0.00) | 1 (1.28) | 7 (8.97) | 25 (32.05) | 45 (57.69) |
| STOP training intervention | 53 | 1 (1.89) | 2 (3.77) | 2 (3.77) | 12 (22.64) | 11 (20.76) | 25 (47.17) |

*of the 134 treated smokers consented to the STOP trial, 3 withdrew at 6 months follow-up

**Table S2: Would you recommend this service to other smokers who want to stop smoking?**

| **Allocation** | **Number of treated smokers** | **No (%)** | **Unsure (%)** | **Yes (%)** | **Missing (%)** |
| --- | --- | --- | --- | --- | --- |
| Control | 78 | 0 (0.00) | 0 (0.00) | 33 (42.31) | 45 (57.69) |
| STOP training intervention | 53 | 1 (1.89) | 0 (0.00) | 27 (50.94) | 25 (47.17) |

**Table S3. Acceptability of the intervention to pharmacy support staff**

|  | **Strongly**  **disagree, % (n)** | **Somewhat**  **disagree,**  **% (n)** | **Neither agree nor disagree, % (n)** | **Somewhat agree,**  **% (n)** | **Strongly agree,**  **% (n)** | **Missing,**  **% (n)** |
| --- | --- | --- | --- | --- | --- | --- |
| I found the training course useful | 0.0% (0) | 2.5% (1) | 5.0% (2) | 25.0% (10) | 47.5% (19) | 20.0% (8) |
| I found the training course enjoyable | 0.0% (0) | 0.0% (0) | 0.0% (0) | 27.5% (11) | 52.5% (21) | 20.0% (8) |
| The training course improved my skills | 0.0% (0) | 0.0% (0) | 2.5% (1) | 32.5% (13) | 45.0% (18) | 20.0% (8) |
| I would recommend the course to others | 0.0% (0) | 0.0% (0) | 7.5% (3) | 15.0% (6) | 57.5% (23) | 20.0% (8) |
| The pharmacy support materials were useful | 0.0% (0) | 5.0% (2) | 5.0% (2) | 32.5% (13) | 37.5% (15) | 20.0% (8) |
| I was able to implement the skills learnt in practice | 0.0% (0) | 5.0% (2) | 10.0% (4) | 30.0% (12) | 35.0% (14) | 20.0% (8) |
| I was able to use social media/WhatsApp easily | 2.5% (1) | 0.0% (0) | 22.5% (9) | 22.5% (9) | 25.0% (10) | 27.5% (11) |

**Table S4: Acceptability of the intervention to pharmacy stop smoking advisors**

|  | **Strongly**  **disagree,**  **% (n)** | **Somewhat**  **disagree,**  **% (n)** | **Neither agree nor disagree, % (n)** | **Somewhat agree,**  **% (n)** | **Strongly agree,**  **% (n)** | **Missing*,**  **% (n)** |
| --- | --- | --- | --- | --- | --- | --- |
| I found the training course useful | 0.0% (0) | 1.85% (1) | 1.85% (1) | 16.67% (9) | 35.19% (19) | 44.44% (24) |
| I found the training course enjoyable | 0.0% (0) | 0.0% (0) | 0.0% (0) | 20.37% (11) | 35.19% (19) | 44.44% (24) |
| The training course improved my skills | 0.0% (0) | 0.0% (0) | 9.26% (5) | 14.82% (8) | 31.48% (17) | 44.44% (24) |
| I would recommend the course to others | 0.0% (0) | 0.0% (0) | 0.0% (0) | 20.37% (11) | 35.19% (19) | 44.44% (24) |
| The pharmacy support materials were useful | 0.0% (0) | 0.0% (0) | 1.85% (1) | 20.37% (11) | 33.33% (18) | 44.44% (24) |
| I was able to implement the skills learnt in practice | 0.0% (0) | 0.0% (0) | 5.56% (3) | 18.52% (10) | 31.48% (17) | 44.44% (24) |
| I was able to use social media/WhatsApp easily | 0.0% (0) | 0.0% (0) | 7.41% (4) | 14.82% (8) | 33.33% (18) | 44.44% (24) |

**Table S5: Stop smoking advisors’ self-efficacy over time**

|  | **STOP training intervention**  **(n = 54)** | | | | **Control**  **(n = 51)** | | | | **treatment group by follow-up**  **interaction term**  **(i.e., treatment effect of interest)** |  |
| --- | --- | --- | --- | --- | --- | --- | --- | --- | --- | --- |
|  | **baseline** | | **follow-up** | | **baseline** | | **follow-up** | |  |  |
|  | **N** | **mean (sd)** | **N** | **mean (sd)** | **N** | **mean (sd)** | **N** | **mean (sd)** |  | **p - value** |
| Self-efficacy: smoker engagement,  recruitment and retention | 52 | 20.54 (3.01) | 39 | 21.85 (2.92) | 50 | 20.58 (3.24) | 43 | 21.28 (3.58) | 0.94 (-0.52 to 2.40) | 0.206 |
| Self-efficacy: behaviour change | 50 | 24.14 (4.20) | 36 | 25.94 (4.67) | 46 | 24.07 (4.50) | 42 | 25.95 (4.38) | 0.49 (-1.51 to 2.51) | 0.628 |
| Self-efficacy: client focused  counselling | 51 | 23.78 (4.23) | 38 | 25.34 (4.12) | 45 | 23.33 (4.31) | 43 | 24.54 (4.50) | 0.67 (-1.21 to 2.54) | 0.485 |
| Self-efficacy: overall | 47 | 69.06 (9.81) | 35 | 73.29 (11.49) | 43 | 68.30 (11.54) | 42 | 71.74 (11.92) | 1.85 (-3.05 to 6.75) | 0.460 |

**Table S6: Pharmacy support staff self-efficacy over time**

|  | **STOP training intervention (n = 40)** | | | | | | **Control (n = 13)** | | | | | |
| --- | --- | --- | --- | --- | --- | --- | --- | --- | --- | --- | --- | --- |
|  | **Baseline** | | | **Follow up** | | | **Baseline** | | | **Follow up** | | |
|  | **N** | **mean**  **(sd)** | **Median (range)** | **N** | **Mean**  **(sd)** | **median**  **(range)** | **N** | **mean**  **(sd)** | **median**  **(range)** | **N** | **Mean**  **(sd)** | **median**  **(range)** |
| Raise the issue of smoking | 40 | 3.4 (1.30) | 3  (1 to 5) | 33 | 4.21 (0.82) | 4  (2 to 5) | 13 | 3.23 (1.17) | 3  (2 to 5) | 7 | 4.29 (0.49) | 4  (4 to 5) |
| Introduce the stop smoking service | 40 | 3.63 (1.33) | 4  (1 to 5) | 33 | 4.18 (1.04) | 5  (1 to 5) | 13 | 3.39 (1.19) | 3  (2 to 5) | 7 | 4.86 (0.38) | 5  (4 to 5) |
| Describing treatment programme | 40 | 3.05 (1.52) | 3  (1 to 5) | 33 | 4.06 (1.14) | 4  (1 to 5) | 13 | 3 (1.23) | 3  (1 to 5) | 7 | 4.71 (0.49) | 5  (4 to 5) |
| Building rapport | 40 | 3.25 (1.30) | 3  (1 to 5) | 33 | 4 (1.17) | 4  (1 to 5) | 13 | 3.39 (1.04) | 3  (2 to 5) | 7 | 4.57 (0.54) | 5  (4 to 5) |
| Engaging clients in service | 39 | 3.13 (1.26) | 3  (1 to 5) | 31 | 4.26 (0.93) | 5  (2 to 5) | 13 | 3.62 (0.87) | 4  (2 to 5) | 7 | 4.57 (0.54) | 5  (4 to 5) |
| Assessing commitment, readiness,  ability to quit | 40 | 2.83 (1.47) | 3  (1 to 5) | 32 | 3.75 (1.14) | 4  (1 to 5) | 13 | 2.92 (1.19) | 3  (1 to 5) | 5 | 4 (1.73) | 5  (1 to 5) |
| Self-efficacy: smoker engagement,  recruitment and retention | 39 | 16.41 (5.78) | 17  (5 to 25) | 31 | 20.68 (3.94) | 22  (10 to 25) | 13 | 16.62 (4.94) | 16  (9 to 25) | 7 | 23 (1.83) | 23  (20 to 25) |
| Self-efficacy: client focused counselling | 40 | 2.83 (1.47) | 3  (1 to 5) | 32 | 3.75 (1.14) | 4  (1 to 5) | 13 | 2.92 (1.19) | 3  (1 to 5) | 5 | 4 (1.73) | 5  (1 to 5) |
| Self-efficacy: building rapport | 40 | 3.25 (1.30) | 3  (1 to 5) | 33 | 4 (1.17) | 4  (1 to 5) | 13 | 3.39 (1.04) | 3  (2 to 5) | 7 | 4.57 (0.54) | 5  (4 to 5) |
| Self-efficacy: overall | 39 | 19.21 (7.03) | 19  (6 to 30) | 30 | 24.5 (4.90) | 27  (11 to 30) | 13 | 19.54 (5.97) | 19  (10 to 30) | 5 | 26.8 (2.59) | 26  (24 to 30) |
